# Retraction: Diffuse Uterine Leiomyomatosis: A Rare Case of Symmetrically Enlarged Uterus

**DOI:** 10.7759/cureus.r169

**Published:** 2025-03-05

**Authors:** Divyajat Kumar, Vishav Bir S Thakur, Suhas M

**Affiliations:** 1 Department of Radiodiagnosis, Dr. D. Y. Patil Medical College, Hospital & Research Centre, Dr. D. Y. Patil Vidyapeeth (Deemed to Be University), Pune, IND

This article has been retracted by the Editors-in-Chief due to image fraud. Figure 5 was presented as original, but was taken from an online source: https://www.sciencephoto.com/media/c0577194/view. The authors agree with the decision to retract.

